# The fatty-acid amide hydrolase inhibitor URB597 inhibits MICA/B shedding

**DOI:** 10.1038/s41598-020-72688-y

**Published:** 2020-09-23

**Authors:** Kazuma Sekiba, Motoyuki Otsuka, Takahiro Seimiya, Eri Tanaka, Kazuyoshi Funato, Yu Miyakawa, Kazuhiko Koike

**Affiliations:** 1 Department of Gastroenterology, Graduate School of Medicine, The University of Tokyo, 7-3-1 Hongo, Bunkyo-ku, Tokyo, 113-8655 Japan; 2 Research Fellow of Japan Society for the Promotion of Science, Tokyo, Japan

**Keywords:** Proteases, Gastrointestinal cancer, Tumour immunology, Tumour virus infections, Hepatitis B virus, Hepatitis C virus, Post-translational modifications

## Abstract

MICA/B proteins are expressed on the surface of various types of stressed cells, including cancer cells. Cytotoxic lymphocytes expressing natural killer group 2D (NKG2D) receptor recognize MICA/B and eliminate the cells. However, cancer cells evade such immune recognition by inducing proteolytic shedding of MICA/B proteins. Therefore, preventing the shedding of MICA/B proteins could enhance antitumor immunity. Here, by screening a protease inhibitor library, we found that the fatty-acid amide hydrolase (FAAH) inhibitor, URB597, suppresses the shedding of MICA/B. URB597 significantly reduced the soluble MICA level in culture medium and increased the MICA level on the surface of cancer cells. The effect was indirect, being mediated by increased expression of tissue inhibitor of metalloproteinases 3 (TIMP3). Knockdown of TIMP3 expression reversed the effect of URB597, confirming that TIMP3 is required for the MICA shedding inhibition by URB597. In contrast, FAAH overexpression reduced TIMP3 expression and the cell-surface MICA level and increased the soluble MICA level. These results suggest that inhibition of FAAH could prevent human cancer cell evasion of immune-mediated clearance.

## Introduction

Major histocompatibility complex class I polypeptide-related sequence A (MICA) and MICB are highly expressed in many infected or transformed human cells. They also tag cells for elimination by natural killer (NK) cells and T cells by activating NK group 2D (NKG2D) receptor^1–3^. We previously identified a single-nucleotide polymorphism in the 5′-flanking region of the MICA gene on 6p21.33 (rs2596452) that leads to the development of hepatocellular carcinoma (HCC) in patients with chronic hepatitis by regulating MICA/B expression^4^. Because MICA/B polymorphisms and expression are critical in human cancers^4–11^, the immune mechanisms mediated by MICA/B ligands and the NKG2D receptor are crucial for antitumor immunity and prevention of carcinogenesis.

The immune-recognition pathway comprising MICA/B and NKG2D is regulated by the proteolytic shedding of MICA/B^12,13^. Although cell-surface MICA/B facilitate the antitumor effects of NK and CD8 T cells via NKG2D receptor activation^14^, pathogenic cells escape by inducing proteolytic shedding of surface MICA/B. In this case, NK and CD8 cells cannot recognize tumor cells due to the low surface expression of MICA/B and the increased serum level of decoy MICA/B fragments^15^. Therefore, inhibition of MICA/B shedding may enhance antitumor immunity and prevent oncogenesis. Although several methods of inhibiting MICA/B shedding have been reported^3,16–18^, none are suitable for clinical use.

While we have reported several methods of increasing MICA protein levels using microRNAs, antisense nucleotides, and the clustered regularly interspaced short palindromic repeats-Cas9 system^19–21^, methods of regulating MICA/B shedding are needed to enhance immunity and develop oncogenesis-prevention strategies. Because drug repositioning enables translation of laboratory discoveries to the clinic^22^, we screened a library of protease inhibitors, most of which are under development for clinical use, to identify inhibitors of MICA/B shedding. The results may enhance NKG2D-related immune-mediated elimination of stressed human cells, such as virus-infected cells, premalignant cells, and cancer cells, by maintaining immune-mediated clearance.

## Results

### Identification of inhibitors of MICA/B shedding

**Figure 1:**
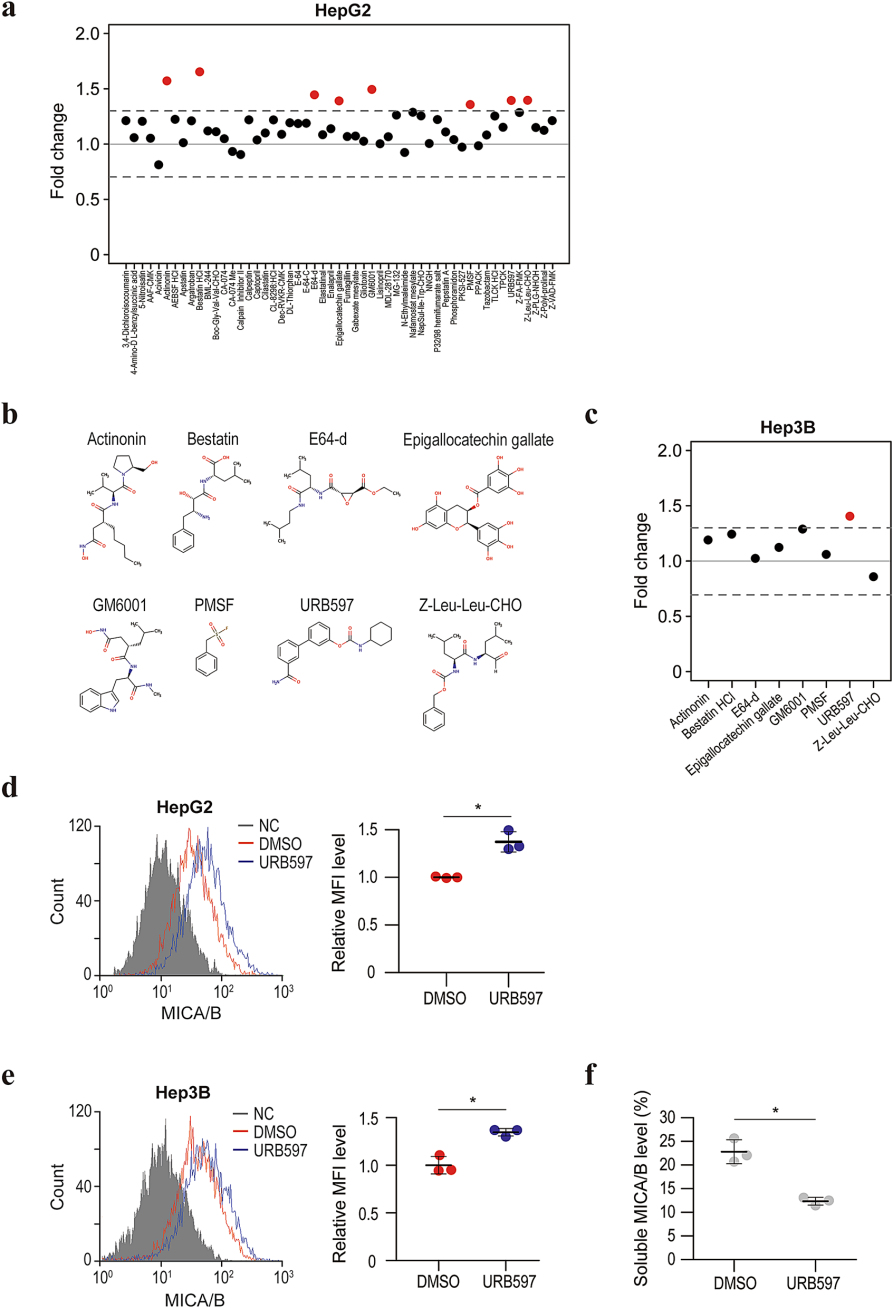
Identification of URB597 as an inhibitor of MICA/B shedding. (**a**) Fold changes in the mean fluorescence intensity (MFI) of surface MICA/B by flow cytometry in HepG2 cells after treatment for 24 h with protease inhibitors. Red, > 1.3-fold change in a biological study. (**b**) Chemical structures of the candidate compounds. (**c**) Fold changes in the MFI of surface MICA/B by flow cytometry in Hep3B cells after treatment for 24 h with the eight candidate protease inhibitors. Red, > 1.3-fold change in a biological study. (**d**) Flow cytometry of surface MICA protein levels in HepG2 cells treated with URB597 (blue line) or DMSO (control, red line). Gray-shaded histograms represent background staining with the isotype IgG. Representative results from three independent experiments are shown. Relative MFI data (*n* = 3) are shown in the right panel. Data are means ± SD. *P = 0.0035 (two-sided Welch’s *t*-test). (**e**) Flow cytometry of surface MICA protein levels in Hep3B cells treated with URB597 (blue line) or DMSO (control, red line). Gray-shaded histograms represent background staining with the isotype IgG. Representative results from three independent experiments are shown. Relative MFI data (*n* = 3) are shown in the right panel. Data are means ± SD. *P = 0.0038 (two-sided Welch’s *t*-test). (**f**) Soluble MICA/B quantification by NanoLuc assay in the culture medium of HepG2 cells treated with URB597 or DMSO. Data are means ± SD from three independent experiments. *P = 0.0025 (two-sided Welch’s *t*-test).

We screened a compound library consisting of 53 protease inhibitors to identify inhibitors of MICA/B shedding. MICA/B expression on the cell surface was determined by flow cytometry in comparison with dimethyl sulfoxide (DMSO) as the control. Using a threshold of a > 30% increase in the cell-surface MICA/B level compared with DMSO in the first screening, we identified eight candidate drugs: actinonin, bestatin, E64-d, epigallocatechin gallate, GM6001, PMSF, URB597, and Z-Leu-Leu-CHO (Fig. 1a,b). Among those compounds, the FAAH inhibitor URB597 had the most reproducible and strongest inhibitory effect on HepG2 and Hep3B cells (Fig. 1c–e). To confirm the inhibition of MICA/B shedding by URB597, we performed a luciferase-based MICA shedding assay^23^. URB597 significantly reduced the soluble MICA level in the culture medium (Fig. 1f). Therefore, URB597 is a candidate inhibitor of MICA/B shedding.

### URB597 inhibits MICA/B shedding via an indirect mechanism

**Figure 2:**
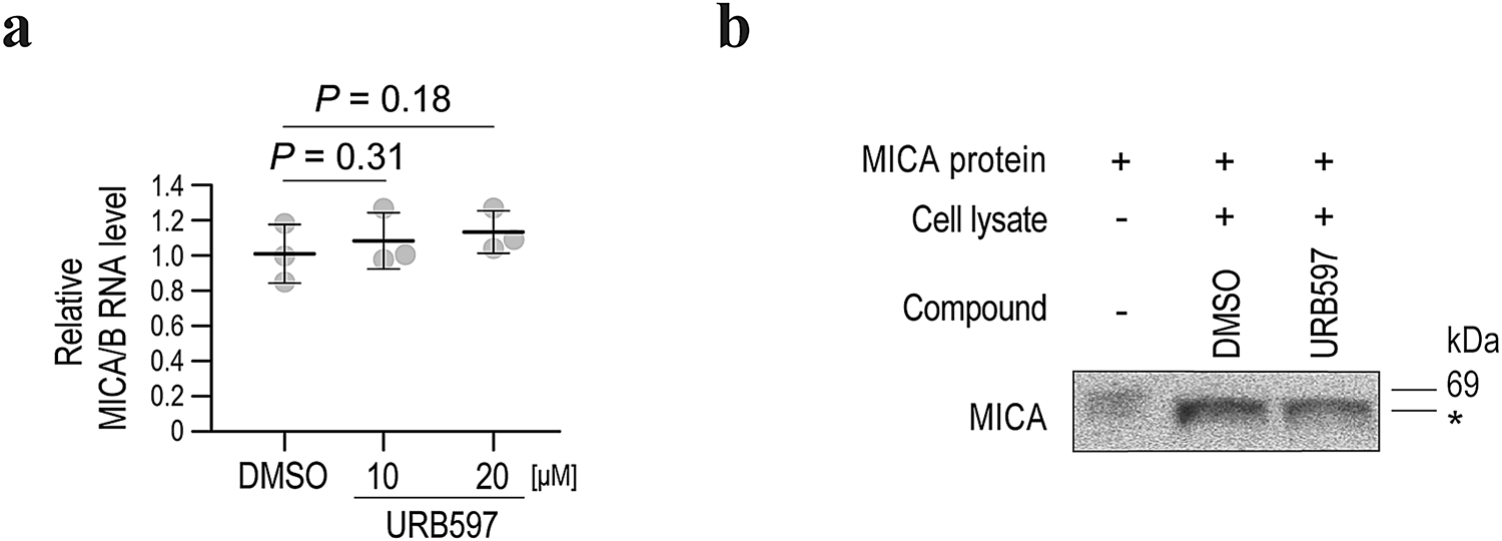
Inhibition of MICA/B shedding by URB597 is an indirect effect. (**a**) qRT-PCR analysis of the MICA/B mRNA levels in HepG2 cells treated with URB597 or DMSO (control). Data are means ± SD from three independent experiments. *P*-values of 0.31 and 0.18 (two-sided Welch’s *t*-test) were not considered to indicate statistical significance. (**b**) In vitro assay of MICA shedding by Western blotting. *Smaller sized recombinant MICA protein, probably reflecting a cleaved form. Representative images of three independent experiments are shown.

To exclude the possibility that URB597 affects the expression of MICA/B, we next assayed the effect of URB597on MICA/B mRNA levels. The MICA/B mRNA levels were not significantly changed by URB597 (Fig. 2a), suggesting that the increase in the cell-surface MICA/B levels caused by URB597 is not due to transcriptional effects.

Next, we performed an in vitro MICA cleavage assay to determine whether URB597 inhibits MICA/B shedding directly or indirectly. Although application of cell lysates with recombinant MICA protein led to the production of smaller sized MICA proteins, suggesting cleavage by proteases (Fig. 2b), addition of URB597 did not affect the size (Fig. 2b). Therefore, the effect of URB597 on MICA/B shedding is indirect and likely mediated by changes in gene expression.

### Tissue inhibitor of metalloproteinases 3 (TIMP3) expression is associated with the effect of URB597 on MICA/B shedding

**Figure 3:**
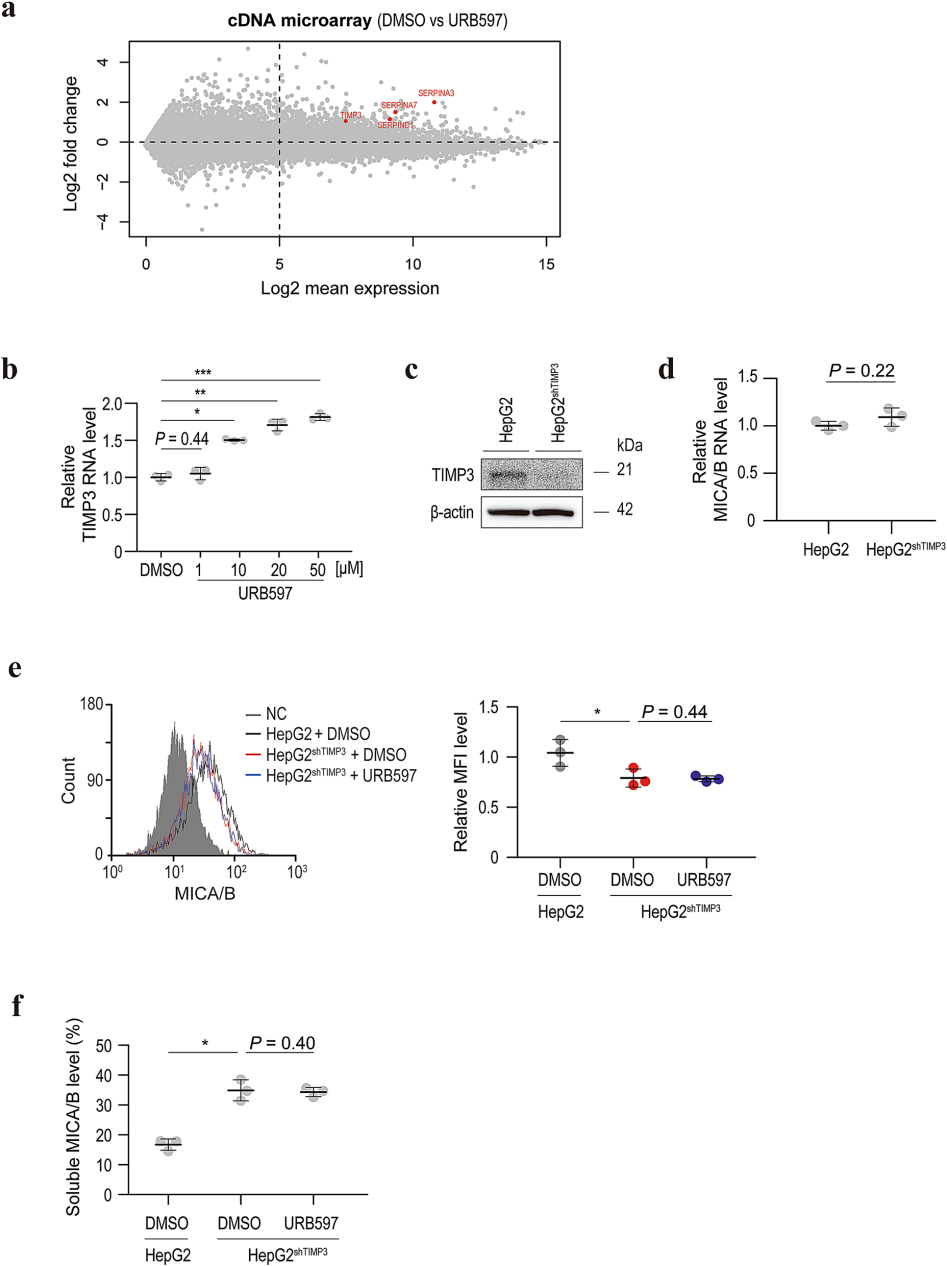
Upregulation of TIMP3 expression by URB597 is responsible for the inhibition of MICA/B shedding. (**a**) A plot of the distribution of data from a cDNA microarray. The X- and Y-axes represent the average values of the log2-converted expression levels and the differences of log2-converted expression levels, respectively. SERPINA3, SERPINA7, SERPIND1, and TIMP3 (red) were identified as candidate inhibitors of MICA/B shedding. (**b**) qRT-PCR analysis of the TIMP3 mRNA level in HepG2 cells after treatment with URB597 or DMSO (control). Data are means ± SD from three independent experiments. A *P*-value of 0.44 (two-sided Welch’s *t*-test) was not considered to indicate significance *P = 7.4 × 10^−5^; *P = 2.0 × 10^−4^; ***P = 3.3 × 10^−5^ (two-sided Welch’s *t*-test). (**c**) Western blots of lysates from HepG2 cells without or with stable TIMP3-specific shRNA expression (HepG2^shTIMP3^). Representative images of three independent experiments are shown. (**d**) qRT-PCR analysis of the MICA/B mRNA levels in HepG2 and HepG2^shTIMP3^ cells. Data are means ± SD from three independent experiments. A *P*-value of 0.22 (two-sided Welch’s *t*-test) was not considered to indicate significance. (**e**) Flow cytometry of surface MICA protein levels in HepG2 cells treated with DMSO (black line) and HepG2^shTIMP3^ cells treated with DMSO (blue line) or URB597 (blue line). Gray-shaded histograms represent background staining with the isotype IgG. Representative results from three independent experiments are shown. Relative MFI data (*n* = 3) are shown in the right panel. Data are means ± SD. A *P*-value of 0.44 (two-sided Welch’s *t*-test) was not considered to indicate significance. *P = 0.027 (two-sided Welch’s *t*-test). (**f**) Soluble MICA/B quantification by NanoLuc assay in the culture medium of HepG2 or HepG2^shTIMP3^ cells treated with DMSO or URB597. Data are means ± SD from three independent experiments. A *P*-value of 0.40 (two-sided Welch’s *t*-test) was not considered to indicate significance. *P = 0.0020 (two-sided Welch’s *t*-test).

Because URB597 did not appear to regulate MICA/B shedding directly, its effect on gene expression was determined using a genome-wide cDNA array. Among the genes upregulated by URB597, we selected SERPINA3, SERPINA7, SERPIND1, and TIMP3 as candidate genes (Fig. 3a), because they are related to proteolytic pathways. Among them, TIMP3 expression was increased by URB597 in a dose-dependent manner (Fig. 3b).

To confirm the involvement of TIMP3 in MICA/B shedding, we established stably TIMP3-specific short-hairpin (sh) RNA-expressing cells (Fig. 3c). Although the MICA/B mRNA levels were not changed in these cells (Fig. 3d), surface MICA/B expression was significantly decreased (Fig. 3e). Moreover, the soluble MICA/B levels were significantly increased by knockdown of TIMP3 (Fig. 3f). Importantly, in TIMP3-knockdown cells, URB597 had no effect on MICA shedding (Fig. 3e,f). Therefore, the increased TIMP3 expression induced by URB597 inhibits MICA/B shedding.

### URB597-induced inhibition of MICA/B shedding is dependent on FAAH

**Figure 4:**
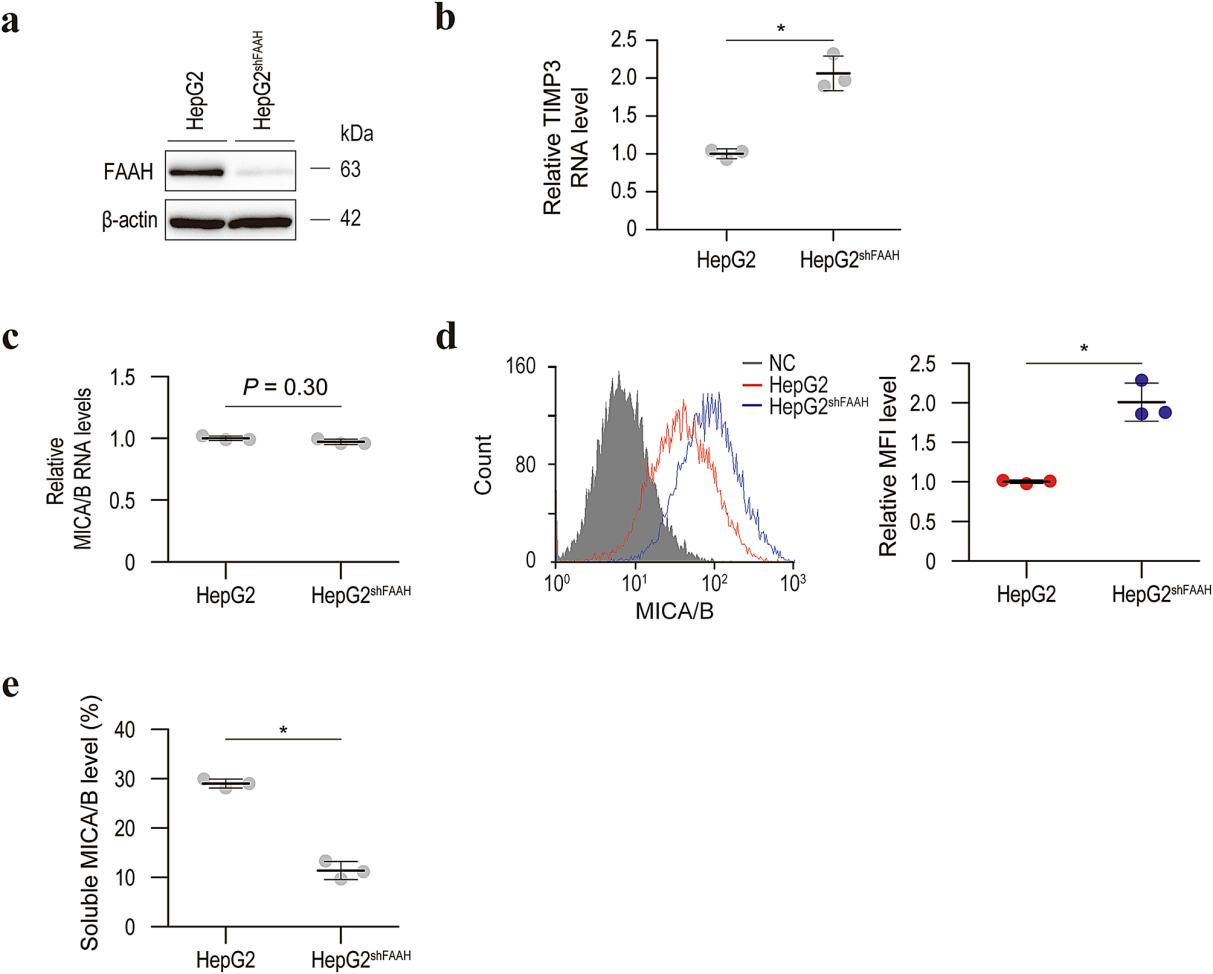
Knockdown of FAAH results in activation of TIMP3 transcription and inhibition of MICA/B shedding. (**a**) Western blot of lysates from HepG2 cells without or with stable expression of FAAH-specific shRNAs (HepG2^shFAAH^). Representative images of three independent experiments are shown. (**b**) qRT-PCR analysis of TIMP3 mRNA levels in HepG2 and HepG2^shFAAH^ cells. Data are means ± SD from three independent experiments. *P = 0.010 (two-sided Welch’s t-test). (**c**) qRT-PCR analysis of MICA/B mRNA levels in HepG2 and HepG2^shFAAH^ cells. Data are means ± SD from three independent experiments. A *P*-value of 0.30 (two-sided Welch’s t-test) was not considered to indicate significance. (**d**) Flow cytometry of surface MICA/B protein levels in HepG2 (red line) and HepG2^shFAAH^ (blue line) cells. Gray-shaded histograms represent background staining with the isotype IgG. Representative results from three independent experiments are shown. Relative MFI data (*n* = 3) are shown in the right panel. Data are means ± SD. *P = 0.018 (two-sided Welch’s t-test). (**e**) Soluble MICA/B quantification by NanoLuc assay in the culture medium of HepG2 and HepG2^shFAAH^ cells. Data are means ± SD from three independent experiments. *P = 7.7 × 10^−4^ (two-sided Welch’s t-test).

**Figure 5:**
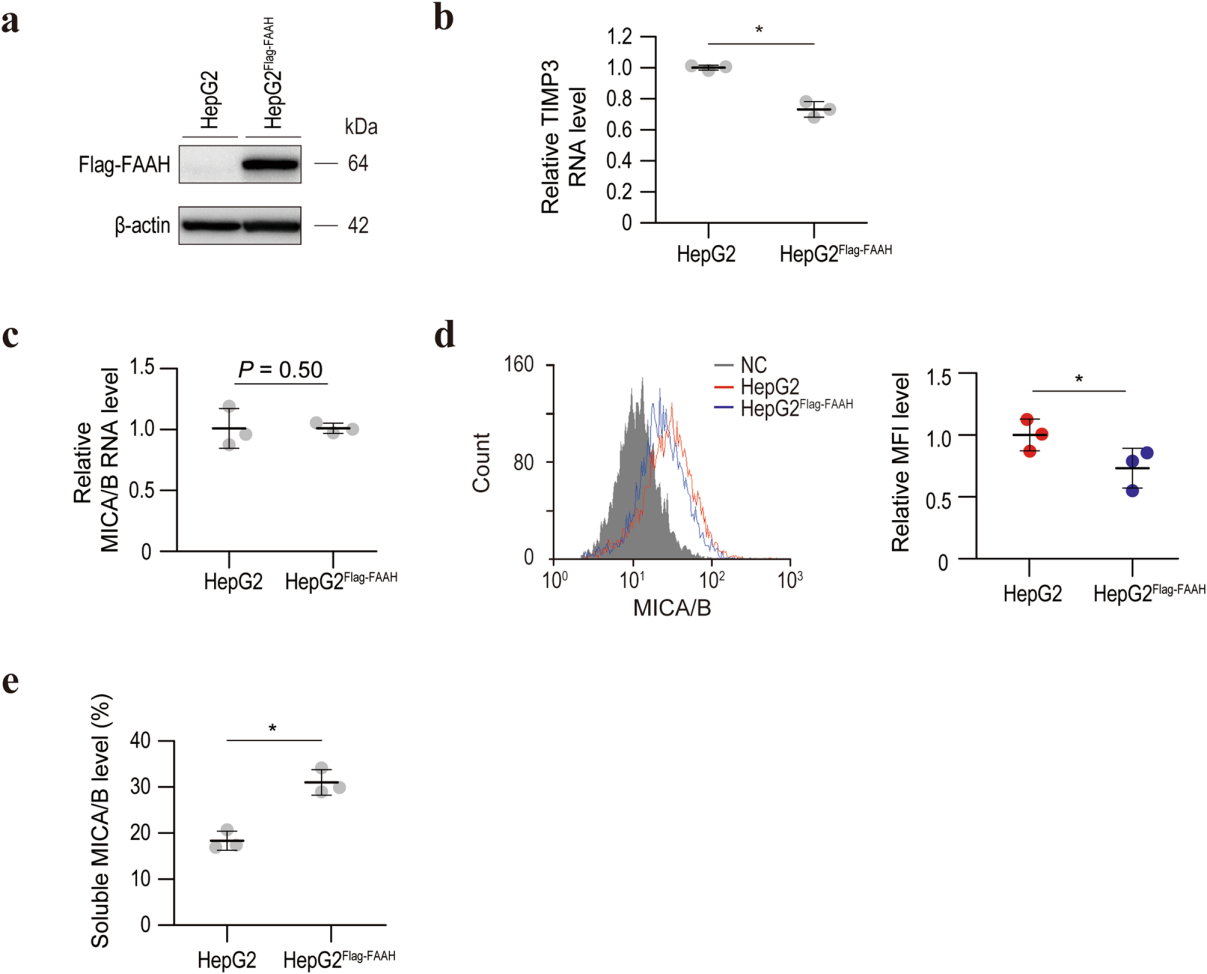
FAAH downregulates TIMP3 expression and promotes MICA/B shedding. (**a**) Western blot of lysates from HepG2 cells without or with stable FLAG-tagged FAAH expression (HepG2^Flag-FAAH^). Representative images of three independent experiments are shown. (**b**) qRT-PCR analysis of the TIMP3 mRNA levels in HepG2 and HepG2^Flag-FAAH^ cells. Data are means ± SD from three independent experiments. *P = 9.1 × 10^−3^ (two-sided Welch’s *t*-test). (**c**) qRT-PCR analysis of the MICA/B mRNA levels in HepG2 and HepG2^Flag-FAAH^ cells. Data are means ± SD from three independent experiments. *P*-value of 0.50 (two-sided Welch’s *t*-test) was not considered as significant. (**d**) Flow cytometry of surface MICA/B protein levels in HepG2 (red line) and HepG2^Flag-FAAH^ (blue line) cells. Gray-shaded histograms represent background staining with the isotype IgG. Representative results from three independent experiments are shown. Relative MFI data (*n* = 3) are shown in the right panel. Data are means ± SD. *P = 0.0043 (two-sided Welch’s *t*-test). (**e**) Soluble MICA/B quantification by NanoLuc assay in the culture medium of HepG2 and HepG2^Flag-FAAH^ cells. Data are means ± SD from three independent experiments. *P = 0.0032 (two-sided Welch’s *t*-test).

Although URB597 is a known FAAH inhibitor and it efficiently suppressed FAAH activity in our experimental setting (Supplementary Figure S1), whether its inhibition of MICA/B shedding is dependent on FAAH is unclear. To examine this, we first established stably FAAH-specific shRNA-expressing HepG2 cells (Fig. 4a). In the same manner as with URB597, FAAH knockdown led to the upregulation of TIMP3 mRNA levels and surface MICA/B protein levels and the downregulation of soluble MICA/B levels without influencing MICA/B transcriptional activities (Fig. 4b–e). The same results were obtained in Hep3B cells (Supplementary Figure S2). Consistent with these results, blocking of FAAH by another inhibitor, PF-3845, yielded similar results (Supplementary Figure S3). We then established stably FLAG-tagged FAAH-expressing cells (Fig. 5a). Interestingly, the TIMP3 mRNA level was significantly decreased in these cells (Fig. 5b). Moreover, although the MICA/B mRNA levels were unchanged (Fig. 5c), the surface MICA/B protein levels were significantly decreased in FLAG-tagged FAAH-overexpressing cells (Fig. 5d), and the soluble MICA/B levels were significantly increased (Fig. 5e). Therefore, FAAH promotes MICA/B shedding at least in part by modulating TIMP3 expression.

### TIMP3 expression and MICA/B shedding are regulated by the endocannabinoid system

**Figure 6:**
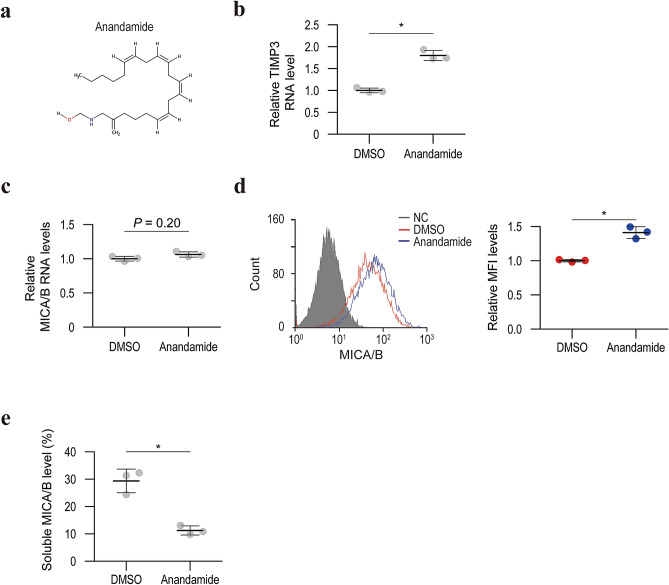
Anandamide downregulates TIMP3 expression and promotes MICA/B shedding. (**a**) The chemical structure of anandamide. (**b**) qRT-PCR analysis of TIMP3 mRNA levels in HepG2 cells with control DMSO or anandamide treatment. Data are means ± SD from three independent experiments. *P = 0.0021 (two-sided Welch’s *t*-test). (**c**) qRT-PCR analysis of the MICA/B mRNA levels in HepG2 cells with control DMSO or anandamide treatment. Data are means ± SD from three independent experiments. *P*-value of 0.20 (two-sided Welch’s *t*-test) was not considered to indicate significance. (**d**) Flow cytometry of surface MICA/B protein levels in HepG2 cells with control DMSO (red line) and anandamide (blue line) treatment. Gray-shaded histograms represent background staining with the isotype IgG. Representative results from three independent experiments are shown. Relative MFI data (*n* = 3) are shown in the right panel. Data are means ± SD. *P = 0.0012 (two-sided Welch’s *t*-test). (**e**) Soluble MICA/B quantification by NanoLuc assay in the culture medium of HepG2 cells with control DMSO or anandamide treatment. Data are means ± SD from three independent experiments. *P = 0.010 (two-sided Welch’s *t*-test).

**Figure 7:**
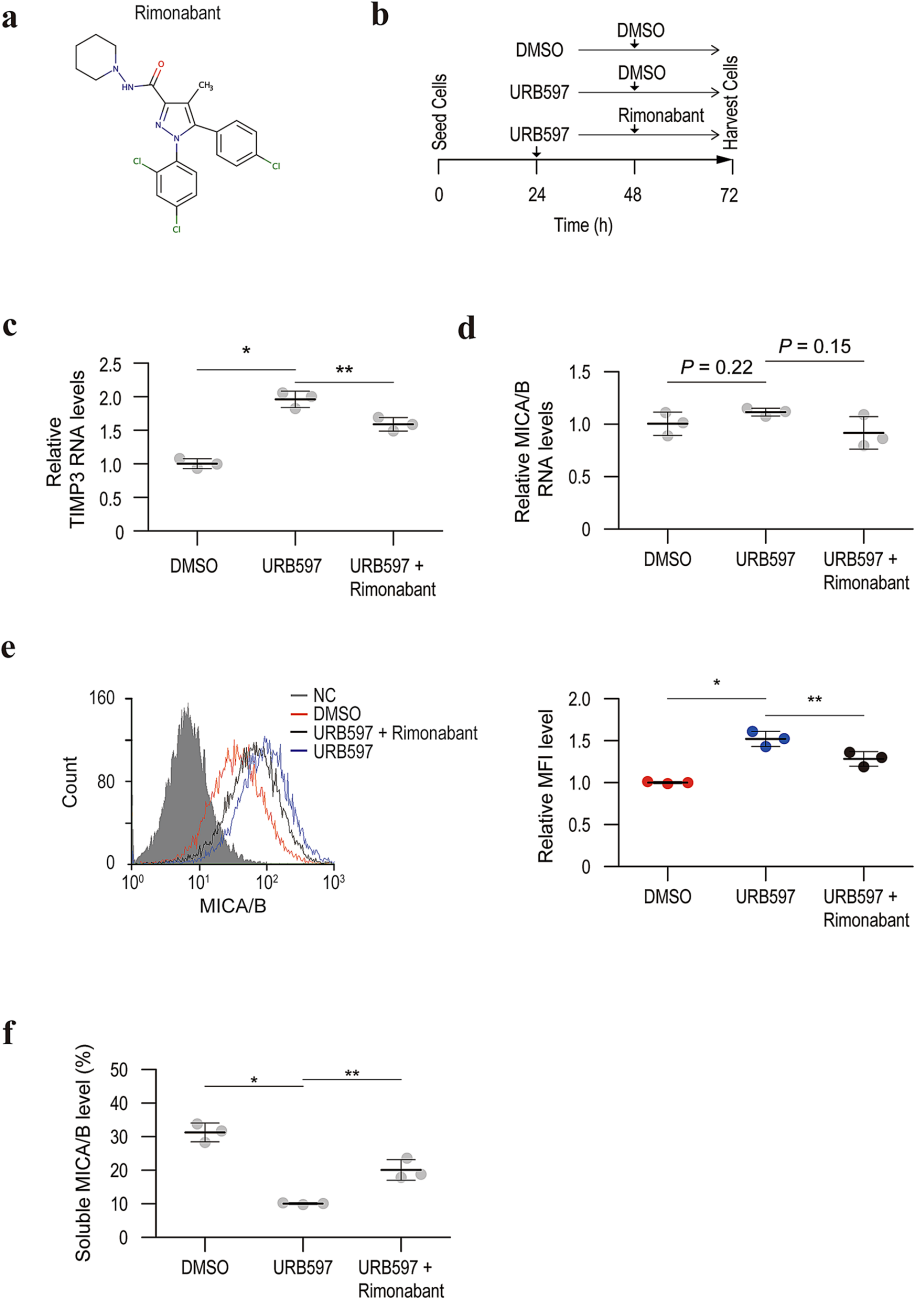
Rimonabant reverses the effect of URB597 on TIMP3 expression and MICA/B shedding. (**a**) The chemical structure of rimonabant, a cannabinoid CB1 receptor antagonist. (**b**) Time course of the experiment. Three groups (control DMSO treatment followed by the addition of control DMSO; URB597 treatment followed by the addition of control DMSO; and URB597 treatment followed by the addition of rimonabant) were analyzed. (**c**) qRT-PCR analysis of TIMP3 mRNA levels in the three experimental groups. Data are means ± SD from three independent experiments. *P = 9.0 × 10^−4^; **P = 0.016 (two-sided Welch’s *t*-test). (**d**) qRT-PCR analysis of the MICA/B mRNA levels in the three experimental groups. Data are means ± SD from three independent experiments. *P*-values of 0.22 and 0.15 (two-sided Welch’s *t*-test) were not considered to indicate significance. (**e**) Flow cytometry of surface MICA/B protein levels in the three experimental groups. Gray-shaded histograms represent background staining with the isotype IgG. Representative results from three independent experiments are shown. Relative MFI data (*n* = 3) are shown in the right panel. Data are means ± SD. *P = 0.0089; **P = 0.031 (two-sided Welch’s *t*-test). (**f**) Soluble MICA/B quantification by NanoLuc assay in the culture medium of the three experimental groups. Data are means ± SD from three independent experiments. *P = 0.0054; **P = 0.029 (two-sided Welch’s *t*-test).

Because FAAH is the primary enzyme responsible for hydrolyzing bioactive amides including anandamide, an agonist of cannabinoid receptor types I and II, TIMP3 activation by URB597 may be involved in activation of the endocannabinoid system. Supporting this hypothesis, anandamide elevated TIMP3 expression levels and cell surface MICA/B levels without influencing the transcriptional activity of MICA/B (Fig. 6). Although URB597 was reported not to increase 2-arachidonoyl glycerol (2-AG) levels^24^, 2-AG is a well-known endocannabinoid and administration of 2-AG resulted in enhanced TIMP3 expression and cell surface MICA/B levels (Supplementary Figure S4). Moreover, a CB1 receptor agonist, AM1241, activated TIMP3 expression and inhibited MICA/B shedding (Supplementary Figure S5). As expected, another CB1 receptor antagonist, rimonabant, partially but significantly attenuated the effect of URB597 on TIMP3 expression and MICA/B shedding (Fig. 7). These results suggest that the increased endocannabinoid is responsible for overexpressing TIMP3 and inhibiting MICA/B shedding.

## Discussion

We found that URB597, an inhibitor of FAAH, suppresses MICA/B shedding by increasing TIMP3 expression. As shown in our genome-wide association study, MICA gene polymorphisms and MICA protein levels are significantly correlated with the incidence of HCC in patients with chronic hepatitis^4^, an effect possibly mediated by modulating the elimination of pre-malignant cells by cytotoxic lymphocytes^1,2^. Therefore, MICA/B-mediated oncoimmunity is crucial in HCC oncogenesis. Tumors, including HCC, evade immune recognition by proteolytic shedding of MICA/B. Although a disulfide isomerase (ERp5), a matrix metalloproteinase (MMP), and a disintegrin and metalloproteinase (ADAM) family member have already been identified as promoters of MICA/B shedding^25–28^, we identified a novel candidate inhibitor of MICA/B shedding. This may be advantageous because a high serum MICA concentration is associated with the progression of several human cancers^29^.

URB597, [3-(3-carbamoylphenyl)phenyl]*N*-cyclohexylcarbamate, is a selective inhibitor of FAAH. FAAH is the primary enzyme that degrades anandamide, an agonist of cannabinoid receptor types I and II. Therefore, inhibition of FAAH results in activation of cannabinoid receptor-mediated intracellular signaling pathways^30^. Indeed, URB597 elevates anandamide levels in vitro and in vivo^30,31^ and exerts antinociceptive and anxiolytic effects in vivo^32^. In addition, cannabinoid-mediated signaling exerts an antitumor effect in several cancers^33–36^. However, the underlying mechanisms are unclear^37^. The inhibition of MICA/B shedding by URB597 may enhance the immune surveillance of cancer cells, which may explain the antitumor effect of cannabinoid-mediated signaling. If so, this would facilitate the development of novel immunotherapeutics for cancer.

The inhibition of MICA/B shedding by URB597 was dependent on TIMP3. Because knockdown of TIMP3 led to increased MICA/B shedding, and URB597 significantly increased TIMP3 expression, URB597 inhibition of MICA/B shedding was likely mediated by increased TIMP3 expression. Because TIMPs (TIMP1, TIMP2, TIMP3, and TIMP4) are inhibitors of MMPs, which induce MICA/B shedding^26,27^, the suppression of MICA/B shedding caused by increased TIMP3 expression may be mediated by MMP inhibition^38^. Indeed, in acute myeloid leukemia cells, the shedding of MICA/B was significantly inhibited by increased expression of TIMP3^38^. Moreover, in patients with acute myeloid leukemia, low TIMP3 expression is significantly associated with an adverse cytogenetic prognosis, possibly due to decreased NK-cell-mediated immune recognition caused by increased shedding of NKG2D ligands^38^. Members of the ADAM family may also mediate shedding of MICA/B^28^. Because it suppresses the function of ADAM10 and ADAM17 in addition to MMPs^39–41^, TIMP3 may regulate MICA/B shedding by regulating MMPs and ADAMs.

The TIMP3 mRNA level was significantly upregulated by URB597. Gene expression is regulated by various mechanisms, such as promoter activity modulation, epigenetic modifications, chromatin regulation, and post-transcriptional mechanisms involving microRNAs. Because FAAH is involved in diverse intracellular signaling^42^, and URB597 may have effects other than FAAH inhibition, the mechanism by which URB597 increases TIMP3 mRNA expression is unclear. Nonetheless, the finding is intriguing, because regulation of MMP function is implicated in a variety of physiological and pathological conditions^43^.

In summary, URB597, an FAAH inhibitor, suppresses MICA/B shedding by increasing TIMP3 expression. Because MICA/B is a regulator of antitumor immunity^1–3^ and the FAAH-mediated pathway is under evaluation as a target for neurological conditions, for example, post-traumatic stress disorder^44,45^, application of FAAH inhibitors in the cancer therapeutic field as a drug repositioning strategy may be a practical method to enhance anti-tumor immunotherapy.

## Methods

### Cells

HEK293T human embryonic kidney cells and HepG2 and Hep3B human hepatocellular carcinoma cells^46^ were purchased from the American Type Culture Collection (Manassas, VA) and cultured in Dulbecco’s modified Eagle’s medium supplemented with 10% fetal bovine serum and 1% penicillin/streptomycin. Cells were incubated at 37 °C in 20% O_2_ and 5% CO_2_.

### Plasmids

The Halo-tagged FAAH-expressing plasmid (#FHC20293) was purchased from Kazusa DNA Research Institute (Chiba, Japan).

The Flag-tagged FAAH-expressing plasmid was constructed by subcloning the Flag-tagged FAAH sequence using the In-fusion method (Clontech, Mountain View, CA). Briefly, after amplifying cDNA using the FAAH-expressing plasmid as the template along with primers containing Flag sequences, the PCR products were cloned into the pCDH-CMV-puro lentivirus vector (System Biosciences, Palo Alto, CA). The primers were 5′-accatggactacaaggatgacgatgacaagatggtgcagtacgagctgtgg-3′ (sense) and 5′-tcaggatgactgcttttcagg-3′ (antisense).

The NanoLuc-conjugated MICA-expressing plasmid was constructed using a two-step cloning method described previously^23^. First, NanoLuc cDNA, amplified by polymerase chain reaction (PCR) using the pNL1.1 NanoLuc vector (Promega, Madison, WI) as the template, was cloned into the *XbaI* site of the pCDH vector (System Biosciences) using the In-fusion method (Clontech). Next, MICA cDNA, PCR amplified from a Halo-tagged MICA expression vector (Promega), was inserted immediately downstream of the NanoLuc cDNA at the *EcoRI* site of the pCDH NanoLuc vector. The following primers were used: 5′-CATAGAAGATTCTAGGCCACCATGGTCTTCACACTC-3′ and 5′-ATTCGCTAGCTCTAGCGCCAGAATGCGTTCGCACAG-3′ for NanoLuc cloning, and 5′-TAGAGCTAGCGAATTCCATGGGGCTGGGCCCGGT-3′ and 5′-ATTTAAATTCGAATTCTTAAACGGCGCCCTCAGTGGA-3′ for MICA cloning.

### Compounds

Screen-well Protease Inhibitor Library BML-2833C version 2.1 was purchased from Enzo Life Sciences (Farmingdale, NY, USA). URB597 was purchased from Cayman Chemical (Ann Arbor, MI, USA); PF-3845, AM1241, and rimonabant were obtained from Selleck Chemicals (Houston, TX, USA); anandamide was obtained from Merck Millipore (Burlington, MA, USA); and 2-AG was obtained from Tocris Bioscience (Bristol, UK). The library includes 53 protease inhibitors, including angiotensin-converting enzyme, aminopeptidase B, calpains, caspases, cathepsins, dipeptidyl peptidase-4, furin, granzyme B, γ-secretase, kallikrein, neutrophil elastase, proteasome, and tripeptidyl peptidase II. Structures of the candidate compounds (Fig. 1b) were drawn using MarvinSketch version 20.11.0 (ChemAxon, Budapest, Hungary). A full list is available on the vendor’s webpage.

### Transfection and lentiviral transduction

Transient transfections were performed using Effectene Transfection Reagent (Qiagen, Hilden, Germany). To generate polyclonal cells with stable Flag-FAAH expression, the Lentivirus Packaging System (System Biosciences) was used according to the manufacturer’s instructions as described previously^47^. Briefly, 1.0 µg Flag-FAAH overexpression vector and 5.0 µg pPACKH1 packaging plasmid mix were transfected into HEK293T cells. After 24 h, the collected culture medium was mixed with one-fifth of its volume of PEG-it Reagent (System Biosciences) and incubated overnight at 4 °C to concentrate the virus. After centrifugation, the pellet was resuspended in 1 × phosphate-buffered saline (PBS). The viruses were transduced into HepG2 cells using polybrene (Santa Cruz Biotechnology, Dallas, TX), followed by selection with 6 mg/mL puromycin to obtain polyclonal cells stably expressing Flag-FAAH.

Lentiviruses encoding TIMP3- and FAAH-specific shRNAs were purchased from Santa Cruz Biotechnology (#sc-37022-V and #sc-106807-V). After transduction into HepG2 or Hep3B cells, stable cell lines expressing TIMP3-specific shRNAs (HepG2^shTIMP3^) and FAAH-specific shRNAs (HepG2^shFAAH^ and Hep3B^shFAAH^) were isolated by puromycin selection as described above.

### Flow cytometry

Flow cytometry was performed as described previously^21^. Briefly, cells were hybridized with anti-MICA (1:500; R&D Systems, Minneapolis, MN) or an isotype control IgG (1:500; R&D Systems) for 40 min at 4 °C. After washing, the cells were incubated with a goat anti-mouse antibody conjugated to Alexa Fluor 488 (1:1,000; Molecular Probes, Eugene, OR) for 20 min. Flow cytometry was performed, and the data were analyzed using Guava Easy Cyte Plus (GE Healthcare, Little Chalfont, UK). Mean fluorescence intensity (MFI) was used to estimate the statistical significance.

### Western blot analysis

Western blotting was performed as described previously^48^. Briefly, cells were lysed in triple detergent lysis buffer (50 mM Tris–HCl [pH 7.4], 150 mM NaCl, 1% NP-40, 0.1% sodium dodecyl sulfate [SDS]), and cellular debris was cleared by centrifugation at 15,000 rpm for 10 min. The lysates were separated on a 10–20% polyacrylamide gel (Fujifilm Wako Pure Chemical, Osaka, Japan) by SDS–polyacrylamide gel electrophoresis, followed by electrical transfer to polyvinylidene difluoride membranes (Merck Millipore). Precision Plus Protein All Blue Protein Standards (Bio-Rad, Hercules, CA) were used to estimate the molecular weight. After blocking in 5% dry milk, the membranes were probed overnight at 4 °C with the appropriate primary antibodies diluted in Immunoshot Reagent 1 (Cosmo Bio Co., Ltd., Tokyo, Japan). Subsequently, the corresponding horseradish peroxidase-conjugated secondary antibodies (GE Healthcare, Little Chalfont, UK) were applied. Bound antibodies were detected using the Immunostar LD reagents (Fujifilm Wako Pure Chemical), and images were acquired by WSE-6100 LuminoGraph I (ATTO, Tokyo, Japan). Antibodies against the following factors were used: MICA (#K0218-3, 1:1,000, Medical & Biological Laboratories, Nagoya, Japan), FAAH (#ab54615, 1:1,000, Abcam, Cambridge, UK), TIMP3 (#5673, 1:1,000, Cell Signaling Technology, Danvers, MA), β-actin (#5125, 1:10,000, Cell Signaling Technology), and FLAG (DYKDDDDK) (#018–22381, 1:1,000, Wako Pure Chemical Industries).

### Quantitative reverse-transcription PCR (qRT-PCR)

Total RNA was extracted from cells using RNeasy with DNase (Qiagen). The extracted RNA was reverse transcribed using SuperScript III First-Strand Synthesis SuperMix (Thermo Fisher Scientific, Waltham, MA). To quantify MICA/B mRNA, qRT-PCR was performed using TaqMan Gene Expression Master Mix (Applied Biosystems, Foster City, CA) and the StepOnePlus Real-Time PCR System (Life Technologies, Carlsbad, CA). The MICA/B mRNA levels were normalized to that of β-actin using the TaqMan Gene Expression Assays Hs00741286_m1 (MICA/B) and Hs99999903_m1 (β-actin). To determine TIMP3 expression, qRT-PCR was performed using SYBR qPCR Mix (Toyobo Co, Osaka, Japan) using the StepOnePlus System. The TIMP3 mRNA level was normalized to that of β-actin. The primers used were as follows: TIMP3, 5′-AGTTACCCAGCCCTATGA-3′ (sense) and 5′-GCAAAGGCTTAAACATCT-3′ (antisense); β-actin, 5′-CTGTGCTACGTCGCCCTGG-3′ (sense) and 5′-GCCACAGGACTCCATGCCC-3′ (antisense).

### NanoLuc MICA shedding assay

A NanoLuc MICA shedding assay was performed as reported previously with slight modifications^23^. Briefly, cells seeded onto 10 cm dishes were transfected with 2.0 µg N-terminal NanoLuc-tagged MICA-expressing plasmid, incubated for 12 h, reseeded onto a 96-well plate, and incubated for another 12 h. After application of URB597 or DMSO as the control, the culture medium was collected and mixed with the luminescent substrate (Nano-Glo Luciferase Assay System; Promega), and luminescence values (Luc_sup_) were measured using the GloMax Detection System (Promega). The cells were washed with PBS, and the luminescent substrate (Promega) was added to determine the luminescence values (Luc_cell_) using the GloMax Detection System. Soluble MICA/B levels were determined as Luc_sup_/(Luc_sup_ + Luc_cell_) to adjust for transfection efficiency.

### In vitro assay of MICA shedding

Recombinant MICA protein purchased from Abnova (Taipei, Taiwan) was mixed with HepG2 cell lysate in radioimmunoprecipitation assay buffer (50 mM Tris [pH 7.4], 150 mM ethylenediaminetetraacetic acid, 1% Triton X-100, 1% sodium deoxycholate, and 0.1% SDS). DMSO (control) or URB597 was added and incubated at 37 °C for 1 h. MICA protein was analyzed by Western blotting as described above.

### Genome-wide cDNA array

HepG2 cells were treated with URB597 (10 µM) or DMSO (control) for 24 h. Next, total RNA was extracted, and reverse transcription was performed as described above. The resulting cDNA (3 µg) was subjected to a highly sensitive 3D-Gene microarray (Toray, Tokyo, Japan). The data and the detailed protocols were deposited in a public database (GEO accession #GSE143194).

### Anandamide enzyme-linked immunosorbent assay (ELISA)

Intracellular anandamide concentrations were determined using a Human Anandamide ELISA Kit (#MBS770230, MyBioSource, San Diego, CA) according to the manufacturer’s instructions. Briefly, cells diluted in PBS were subjected to repeated freeze–thaw cycles to release the internal components, followed by centrifuging at 3000 rpm for 20 min. Then, the collected supernatants were added into wells pre-coated with anti-anandamide antibody and mixed with HRP-conjugated streptavidin to form an immune complex. After adding the color developing substrates, the optical density (OD) of each well was measured by a microplate reader (Multiskan FC, Thermo Fisher Scientific) set to 450 nm. The concentration of anandamide was calculated according to the concentration of the standard and the corresponding OD values.

### Statistical analysis

No statistical method was applied to determine the required sample size. Statistical analysis was conducted using R version 3.3.2 (R Foundation for Statistical Computing, Vienna, Austria). Continuous variables are reported as means ± standard deviation (SD) unless indicated otherwise. Welch’s *t*-test was used for comparisons of continuous variables. *P*-values < 0.05 were considered indicative of statistical significance. The experiments were not randomized, and the investigators were not blinded to the group allocation during the experiments or the assessment of outcomes.

## Supplementary information

Supplementary Information 1.

Supplementary Information 2.

Supplementary Information 3.

Supplementary Information 4.

Supplementary Information 5.

Supplementary Information 6.

Supplementary Information 7.

## Data Availability

The authors state that all data supporting the findings of this study are provided within the article and its supplementary files or are available from the authors upon reasonable request.

## Abbreviations

NKG2D: Natural killer group 2D
FAAH: Fatty-acid amide hydrolase
TIMP3: Tissue inhibitor of metalloproteinases 3
MICA: Major histocompatibility complex class I polypeptide-related sequence A
NK: Natural killer
HCC: Hepatocellular carcinoma
DMSO: Dimethyl sulfoxide
MMP: Matrix metalloproteinase
ADAM: A disintegrin and metalloproteinase
PCR: Polymerase chain reaction
PBS: Phosphate-buffered saline
qRT-PCR: Quantitative reverse-transcription PCR
SD: Standard deviation
