# Supplementary material for: The fatty-acid amide hydrolase inhibitor URB597 inhibits MICA/B shedding
Source: Sci Rep. 2020 Sep 23;10:15556. doi: 10.1038/s41598-020-72688-y (PMC7512021; doi:10.1038/s41598-020-72688-y)
Supplement: Supplementary file 1 — Supplementary Information 1. [file 41598_2020_72688_MOESM1_ESM.docx]

**Supplementary Information**

**The fatty-acid amide hydrolase inhibitor URB597 inhibits MICA/B shedding**

Kazuma Sekiba, Motoyuki Otsuka, Takahiro Seimiya, Eri Tanaka, Kazuyoshi Funato,

Yu Miyakawa, and Kazuhiko Koike

**Inventory of Supplementary Information:**

**Supplementary Figure S1~S6 with legends**

**SUPPLEMENTARY FIGURE LEGENDS**

**Supplementary Figure S1. Changes in intracellular anandamide levels by URB597.**

Concentration of anandamide in HepG2 (**a**) and Hep3B (**b**) with control DMSO or URB597 treatment. Data are means ± SD from three independent experiments. *, *P*= 0.0098; **, *P*= 0.011 (two-sided Welch’s *t*-test).

**Supplementary Figure S2. Knockdown of FAAH in Hep3B cells yields results similar to those in HepG2 cells.**

**a**, Western blot of lysates from Hep3B cells without or with stable expression of FAAH-specific shRNAs (Hep3B^shFAAH^). Representative images of three independent experiments are shown. **b**, qRT-PCR analysis of TIMP3 mRNA levels in Hep3B and Hep3B^shFAAH^ cells. Data are means ± SD from three independent experiments. *, *P* = 5.6 × 10^−4^ (two-sided Welch’s *t*-test). **c**, qRT-PCR analysis of MICA/B mRNA levels in Hep3B and Hep3B^shFAAH^ cells. Data are means ± SD from three independent experiments. A *P*-value of 0.98 (two-sided Welch’s *t*-test) was not considered to indicate significance. **d**, Flow cytometry of surface MICA/B protein levels in Hep3B (red line) and Hep3B^shFAAH^ (blue line) cells. Gray-shaded histograms represent background staining with the isotype IgG. Representative results from three independent experiments are shown. Relative MFI data (*n*= 3) are shown in the right panel. Data are means ± SD. *, *P*= 0.020 (two-sided Welch’s *t*-test). **e**, Soluble MICA/B quantification by NanoLuc assay in the culture medium of Hep3B and Hep3B^shFAAH^ cells. Data are means ± SD from three independent experiments. *, *P* = 0.017 (two-sided Welch’s *t*-test).

**Supplementary Figure S3. PF-3845 stimulates TIMP3 mRNA expression and inhibits MICA/B shedding.**

**a**, Chemical structure of PF-3845. **b**, qRT-PCR analysis of TIMP3 mRNA levels in HepG2 cells with control DMSO or PF-3845 treatment. Data are means ± SD from three independent experiments. *, *P*= 0.013 (two-sided Welch’s *t*-test). **c**, qRT-PCR analysis of MICA/B mRNA levels in HepG2 cells with control DMSO or PF-3845 treatment. Data are means ± SD from three independent experiments. A *P*-value of 0.70 (two-sided Welch’s *t*-test) was not considered to indicate significance. **d**, Flow cytometry of surface MICA/B protein levels in HepG2 cells with control DMSO (red line) and PF-3845 (blue line) treatment. Gray-shaded histograms represent background staining with the isotype IgG. Representative results from three independent experiments are shown. Relative MFI data (*n*= 3) are shown in the right panel. Data are means ± SD. *, *P*= 0.0081 (two-sided Welch’s *t*-test). **e**, Soluble MICA/B quantification by NanoLuc assay in the culture medium of HepG2 cells with control DMSO or PF-3845 treatment. Data are means ± SD from three independent experiments. *, *P* = 0.0017 (two-sided Welch’s *t*-test).

**Supplementary Figure S4. 2-arachidonoyl glycerol (2-AG) stimulates TIMP3 mRNA expression and inhibits MICA/B shedding.**

**a**, Chemical structure of 2-AG. **b**, qRT-PCR analysis of TIMP3 mRNA levels in HepG2 cells with control DMSO or 2-AG treatment. Data are means ± SD from three independent experiments. *, *P* = 7.7 × 10^−6^ (two-sided Welch’s *t*-test). **c**, qRT-PCR analysis of MICA/B mRNA levels in HepG2 cells with control DMSO or 2-AG treatment. Data are means ± SD from three independent experiments. A *P*-value of 0.68 (two-sided Welch’s *t*-test) was not considered to indicate significance. **d**, Flow cytometry of surface MICA/B protein levels in HepG2 cells with control DMSO (red line) and 2-AG (blue line) treatment. Gray-shaded histograms represent background staining with the isotype IgG. Representative results from three independent experiments are shown. Relative MFI data (*n* = 3) are shown in the right panel. Data are means ± SD. *, *P*= 0.0010 (two-sided Welch’s *t*-test). **e**, Soluble MICA/B quantification by NanoLuc assay in the culture medium of HepG2 cells with control DMSO or 2-AG treatment. Data are means ± SD from three independent experiments. *, *P*= 0.043 (two-sided Welch’s *t*-test).

**Supplementary Figure S5. AM1241 stimulates TIMP3 mRNA expression and inhibits MICA/B shedding.**

**a**, Chemical structure of AM1241. **b**, qRT-PCR analysis of TIMP3 mRNA levels in HepG2 cells with control DMSO or AM1241 treatment. Data are means ± SD from three independent experiments. *, *P*= 0.0047 (two-sided Welch’s *t*-test). **c**, qRT-PCR analysis of MICA/B mRNA levels in HepG2 cells with control DMSO or AM1241 treatment. Data are means ± SD from three independent experiments. A *P*-value of 0.78 (two-sided Welch’s *t*-test) was not considered to indicate significance. **d**, Flow cytometry of surface MICA/B protein levels in HepG2 cells with control DMSO (red line) and AM1241 (blue line) treatment. Gray-shaded histograms represent background staining with the isotype IgG. Representative results from three independent experiments are shown. Relative MFI data (*n*= 3) are shown in the right panel. Data are means ± SD. *, *P*= 0.0010 (two-sided Welch’s *t*-test). **e**, Soluble MICA/B quantification by NanoLuc assay in the culture medium of HepG2 cells with control DMSO or AM1241 treatment. Data are means ± SD from three independent experiments. *, *P* = 0.0070 (two-sided Welch’s *t*-test).

**Supplementary Figure S6. Full-length blot images for blotting results.**

Full-length western blotting images for Figure 2b (**a**), 3c (**b**), 4a (**c**), 5a (**d**), and S2a (**e**) are shown.
