# Supplementary figures and images for: The fatty-acid amide hydrolase inhibitor URB597 inhibits MICA/B shedding
Source: Sci Rep. 2020 Sep 23;10:15556. doi: 10.1038/s41598-020-72688-y (PMC7512021; doi:10.1038/s41598-020-72688-y)

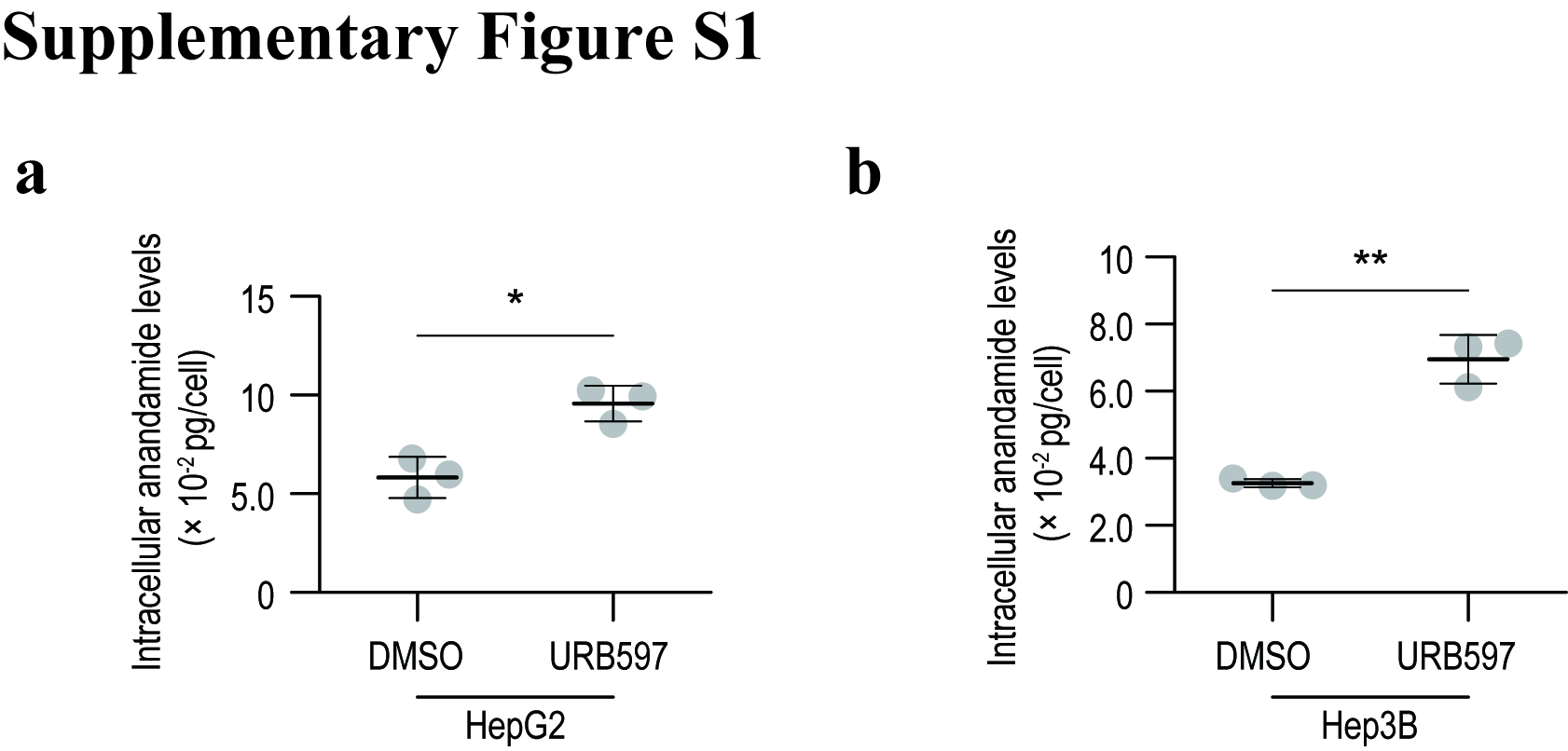

Supplement: Supplementary file 2 — Supplementary Information 2. [file 41598_2020_72688_MOESM2_ESM.tif]

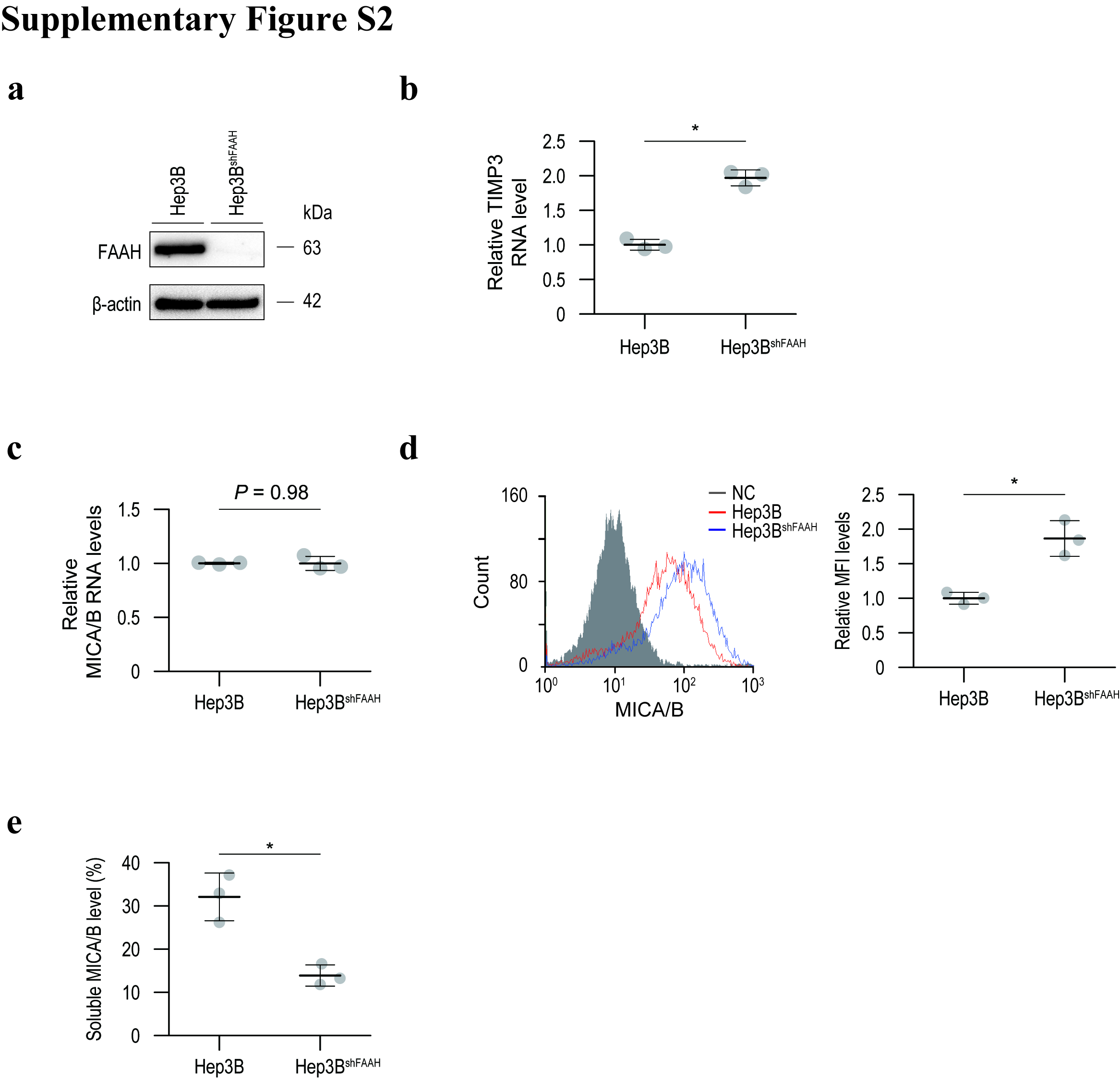

Supplement: Supplementary file 3 — Supplementary Information 3. [file 41598_2020_72688_MOESM3_ESM.tif]

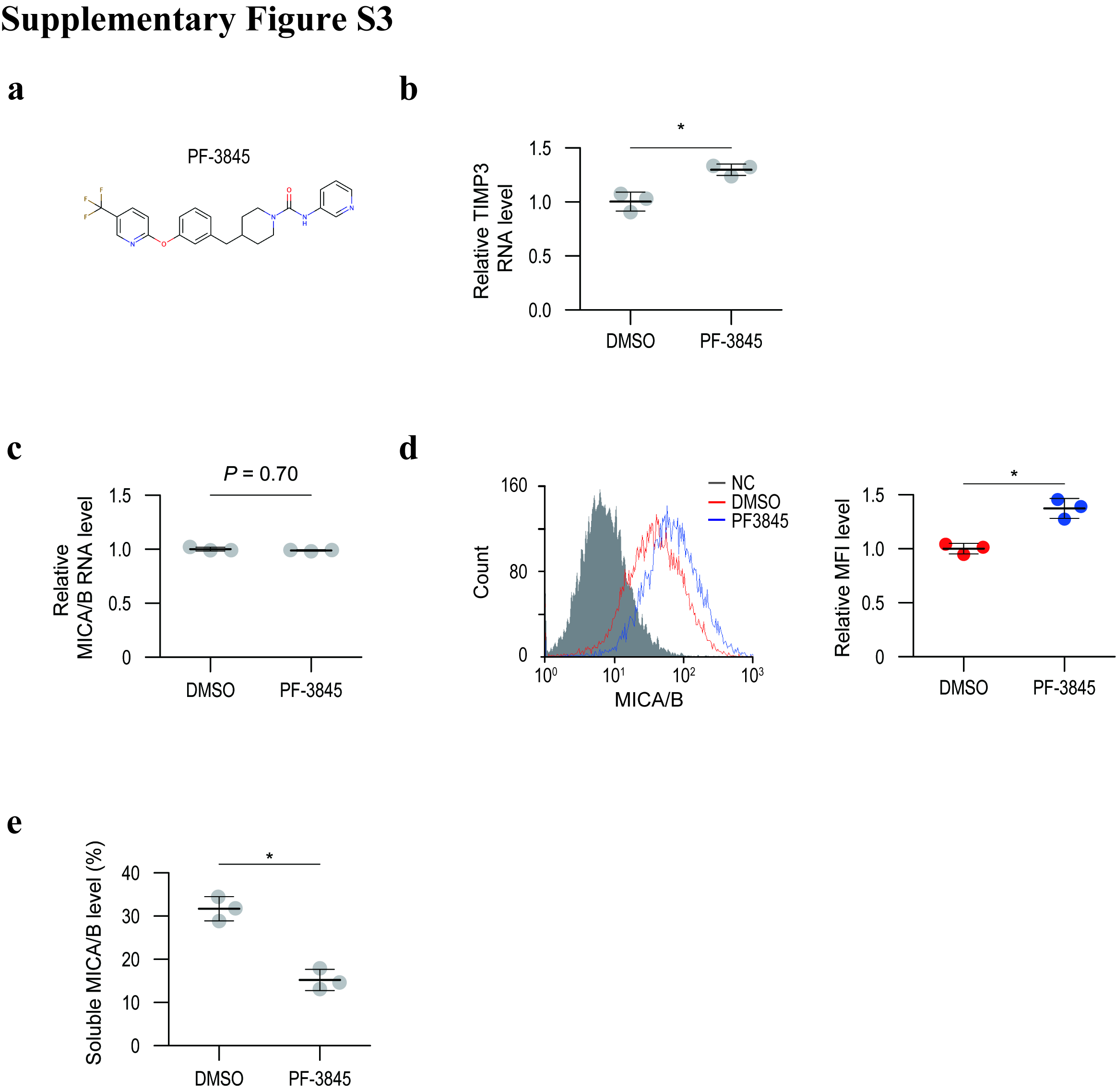

Supplement: Supplementary file 4 — Supplementary Information 4. [file 41598_2020_72688_MOESM4_ESM.tif]

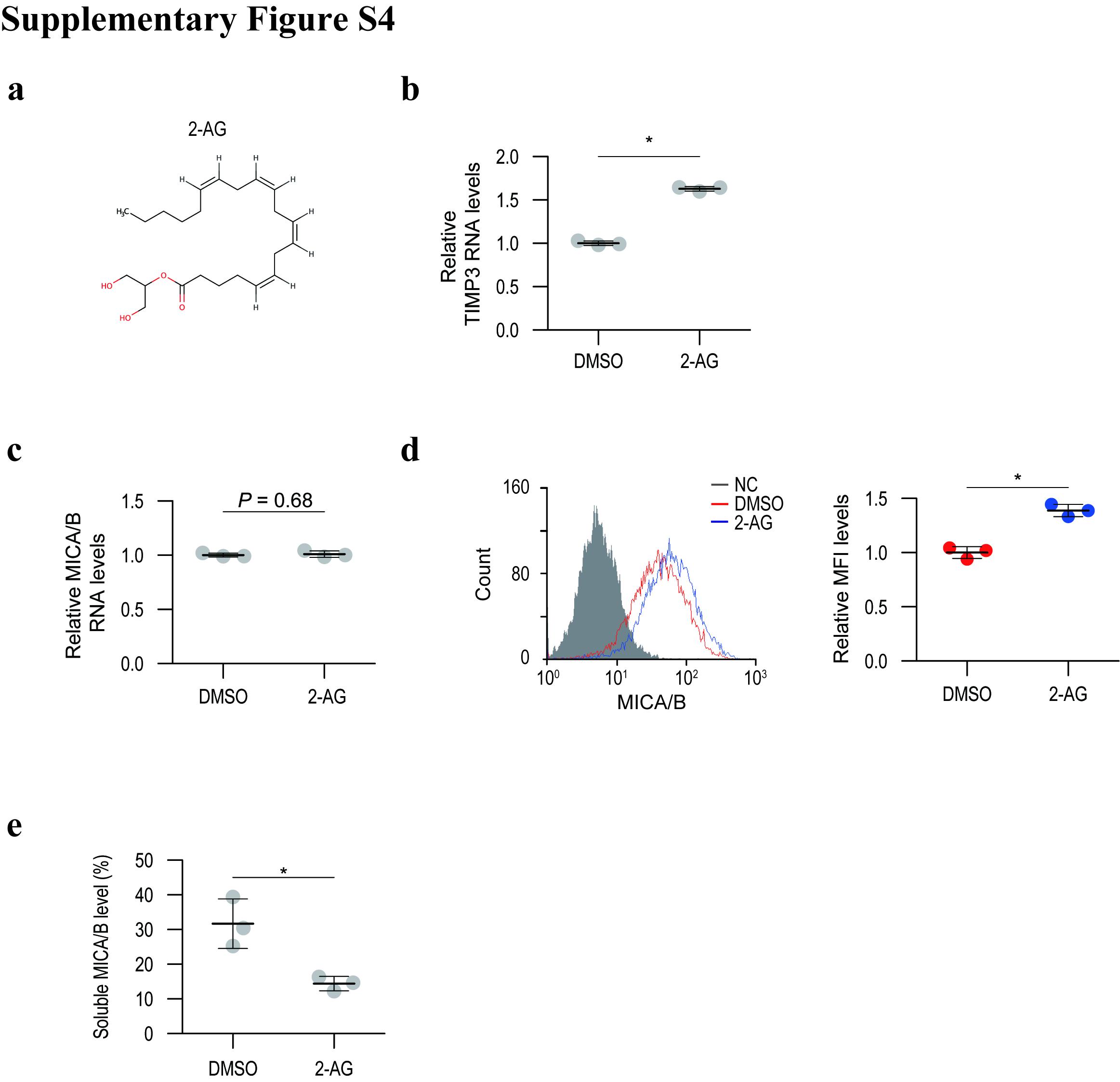

Supplement: Supplementary file 5 — Supplementary Information 5. [file 41598_2020_72688_MOESM5_ESM.tif]

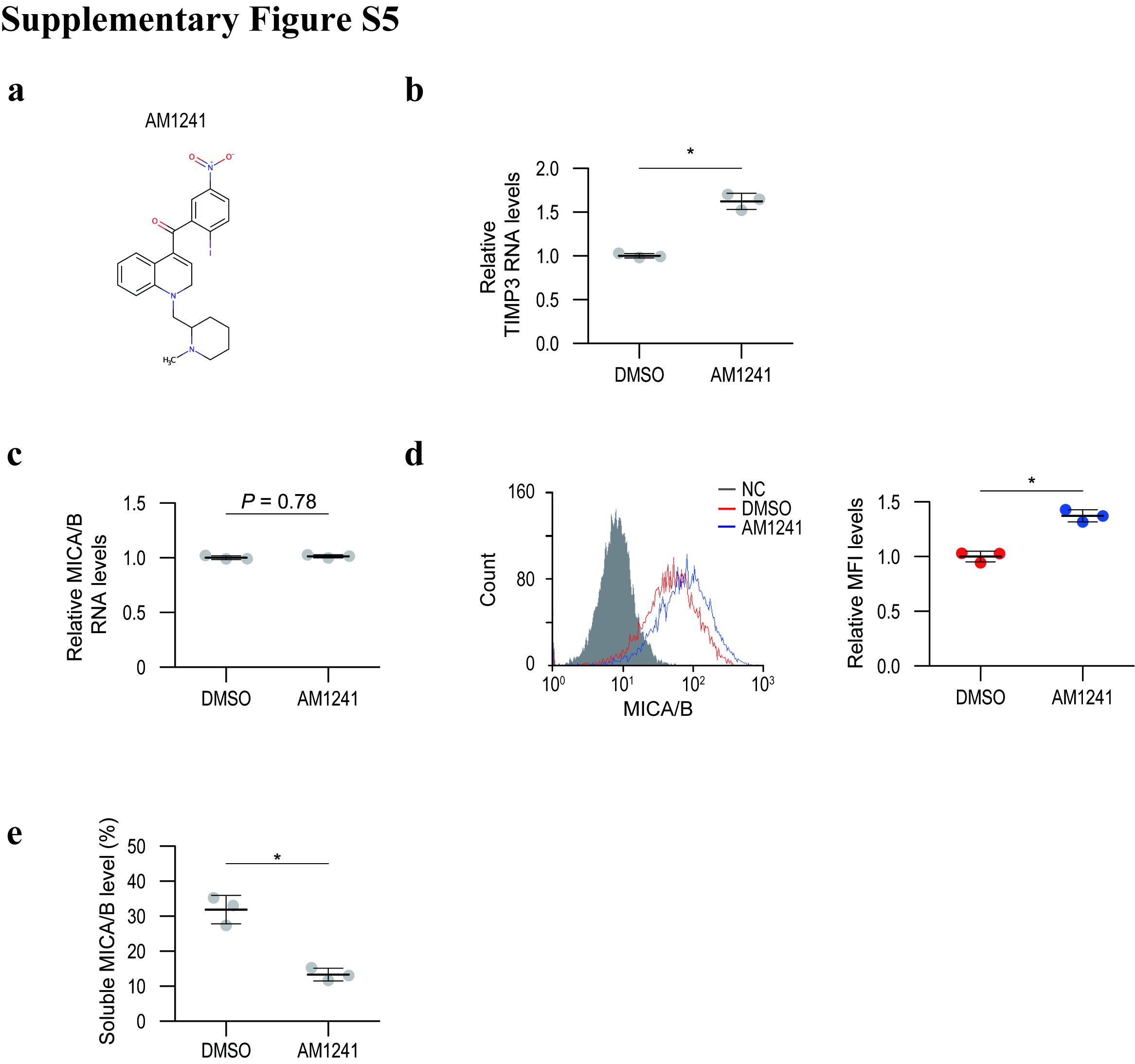

Supplement: Supplementary file 6 — Supplementary Information 6. [file 41598_2020_72688_MOESM6_ESM.tif]

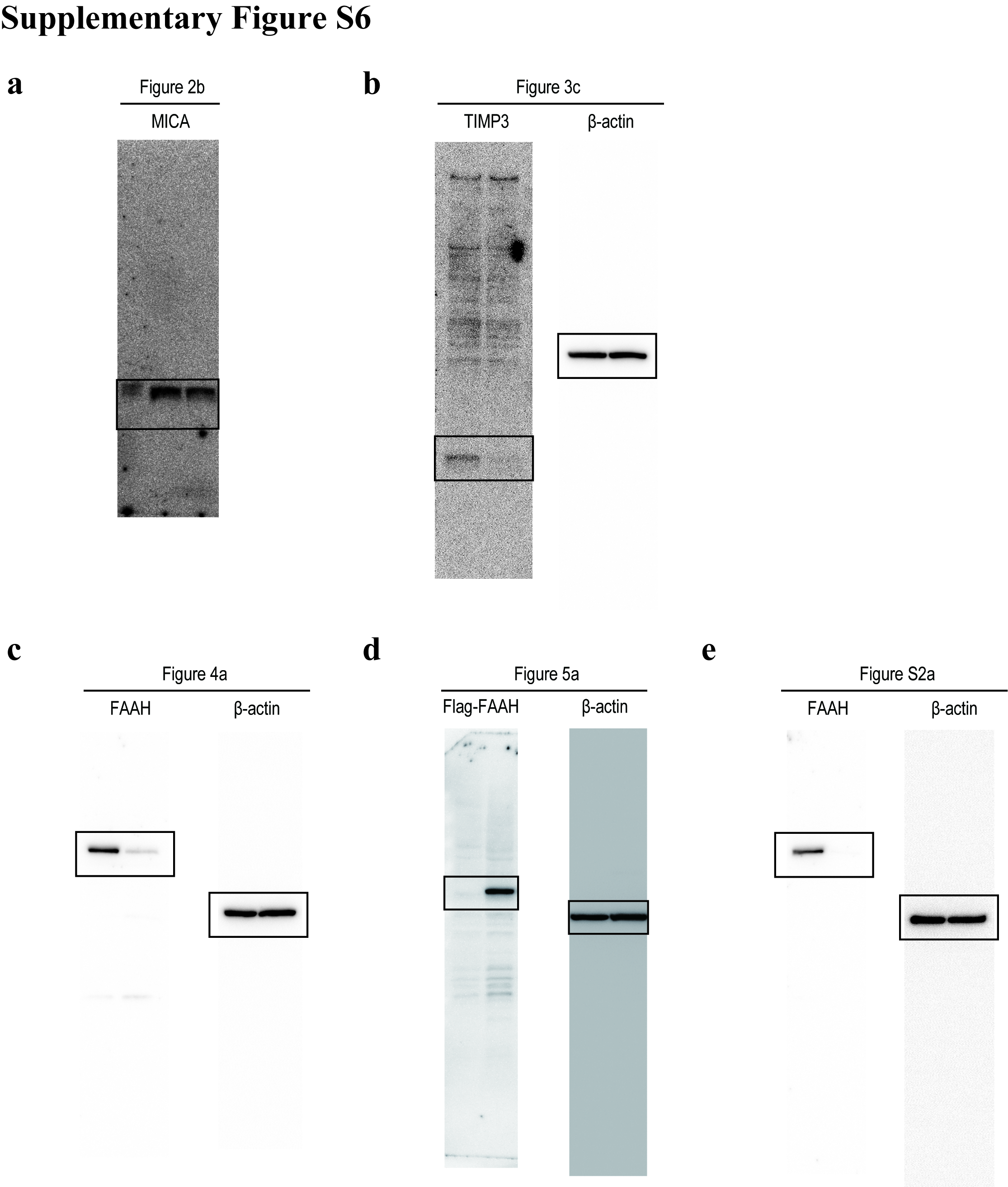

Supplement: Supplementary file 7 — Supplementary Information 7. [file 41598_2020_72688_MOESM7_ESM.tif]
